# Characterization of the plastome of *Camellia pingguoensis* (Theaceae), an endangered and endemic yellow camellia species in China

**DOI:** 10.1080/23802359.2020.1828000

**Published:** 2020-10-07

**Authors:** Xing Wu, Fei-Long He, Hai-Fa Pan, Zu-Lin Ning

**Affiliations:** a Guangdong Provincial Key Laboratory of Applied Botany, South China Botanical Garden, Chinese Academy of Sciences, Guangzhou, China; b Horticulture Research Institute, Anhui Academy of Agriculture Science, Hefei, China

**Keywords:** Yellow camellia, *Camellia pingguoensis*, plastid genome, phylogeny

## Abstract

*Camellia pingguoensis* D. Fang is a shrub which is found on limestone of karst forests in Guangxi, China. In this study, we characterized the whole plastid genome of *C. pingguoensis* using Illumina paired-end sequencing reads. The plastome is 156,621 bp in length, containing two copies of inverted repeat (IR) regions (26,046 bp), a large-single copy (LSC) region (86,289 bp), and a small-single copy (SSC) region (18,240 bp). A total of 114 unique genes in the genome has 80 protein-coding genes, 30 tRNA genes, and 4 rRNA genes. The phylogenetic result indicates *C. pingguoensis* is closely related to *C. nitidissima* C. W. Chi.

*Camellia* L., comprising more than 200 species, is the type and the largest and economically most important genus in the family Theaceae (Vijayan et al. 2009; Huang et al. 2014). Most species of *Camellia* are distributed in southeastern and eastern Asia, China is the center of species diversity, possess more than 80% of the species (Gao et al. 2005). About 82% the species of the genus inhabit the subtropical (the subtropical evergreen broadleaved forests) EBLFs of East Asia (Yu et al. 2017). *Camellia pingguoensis* D. Fang is a shrub which grows between 1-3 meters tall with pale yellow flowers which fade to white when fully opened. It is found on limestone of karst forests between 100–500 meters above sea level in Guangxi (Wheeler 2015). This species is a valuable ornamental plant, and is classified as Endangered in the 2004 China Red List due to continuing population decline and limited distribution (Wheeler 2015). In this study, we characterized the whole plastid genome of *C. pingguoensis*.

Fresh leaves of an individual of *C. pingguoensis* was collected from South China Botanical Garden (23°10′58″N, 113°21′28″E), and used to extract total genome DNA using a modified CTAB protocol (Doyle and Doyle 1987). The voucher specimen was deposited at South China Botanical Garden Herbarium (voucher number: Wu19017). The Illumina paired-end library was prepared and sequenced based on Illumina Hiseq X Ten platform at Beijing Genomics Institute (Wuhan, China). The plastome was assembled using NOVOPlasty (Dierckxsens et al. 2017) with a reference plastome *Camellia granthamiana* Sealy (NC_038181). The draft plastid genome was checked by remapping reads in Geneious Prime 2019 (Biomatters, Ltd, Auckland, New Zealand). The coding genes were annotated and manually adjusted using Geneious Prime 2019, and tRNA were annotated using ARAGORN (Laslett and Canback 2004). Phylogenomic analysis was performed using protein-coding genes from 28 *Camellia* taxa and four outgroups in Theaceae. The aligned matrix was implemented in MAFFT (Katoh and Standley 2013). The maximum likelihood tree was constructed using RAxML (Stamatakis 2014) with 1000 rapid bootstrap replicates (Figure 1).

**Figure 1.**
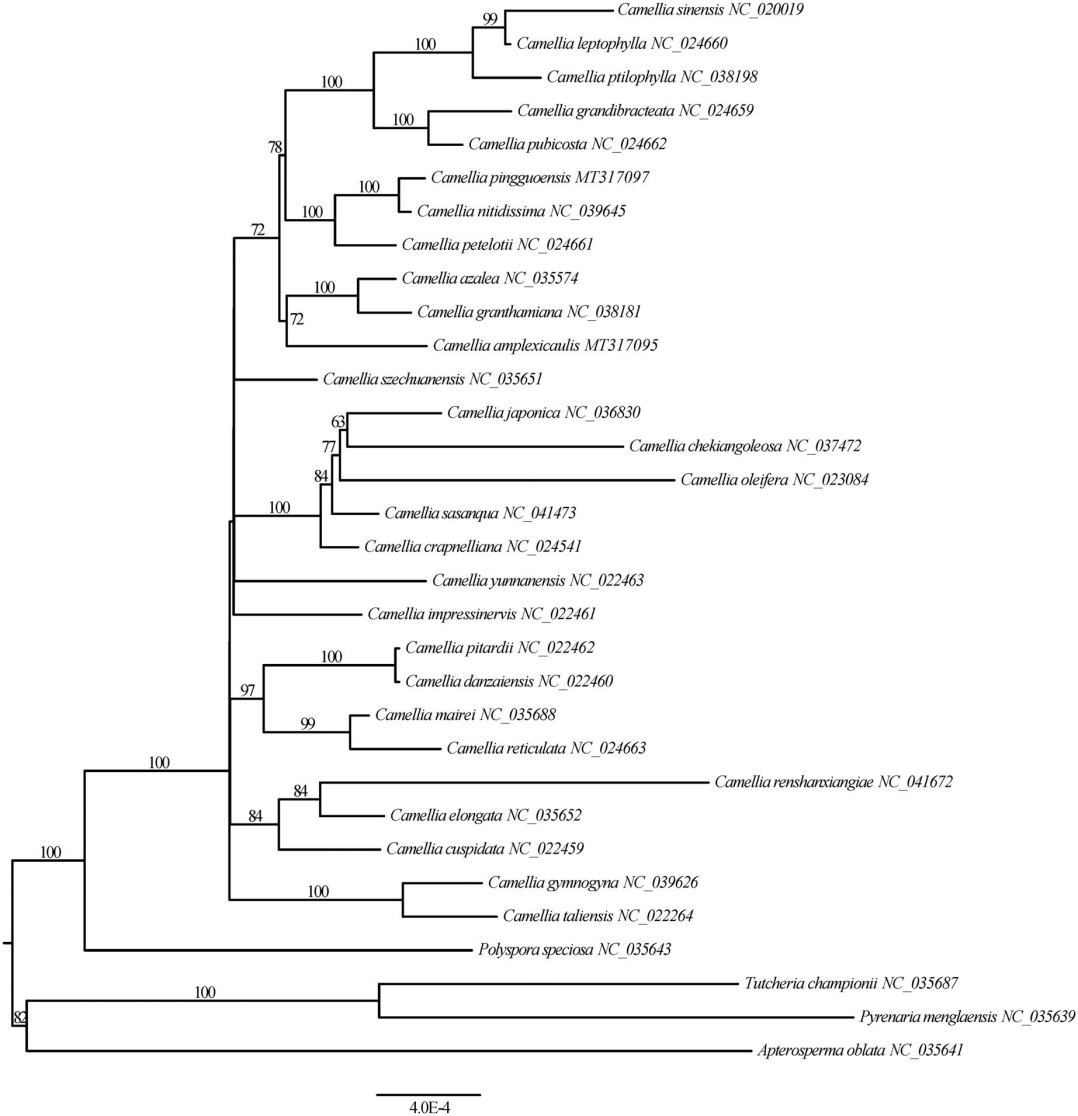
Maximum likelihood tree of *Camellia* based on 79 common protein-coding genes. The node labels indicate the ML bootstrap (1000 replicates) support values.

The plastome of *C. pingguoensis* (GenBank accession number MT317097) is 156,621 bp in length, consisting of two copies of inverted repeat (IR) regions (26,046 bp), a large-single copy (LSC) region (86,289 bp), and a small-single copy (SSC) region (18,240 bp). The plastome has a total of 114 unique genes, including 80 protein-coding genes, 30 tRNA genes, and four rRNA genes. The GC values of the LSC, SSC, and IR regions are 35.4, 30.6, and 43%, respectively. The overall GC content of the plastome is 37.3%. The ML tree showed *C. pingguoensis* and *Camellia nitidissima* C. W. Chi were recovered as sister relationship with strong bootstrap support (100%).

## Data Availability

The data that support the findings of this study are openly available in GenBank at https://www.ncbi.nlm.nih.gov/, reference number MT317097.
